# ‘Playing to extinction’: the commercial determinants of gambling-related harm, suicidality and suicide

**DOI:** 10.1016/j.lanwpc.2025.101685

**Published:** 2025-09-26

**Authors:** Angela Rintoul, Suzanne McLaren, Kerrie Shandley, Britt Klein

**Affiliations:** a Centre for Mental Health and Community Wellbeing, University of Melbourne, Australia; b Health Innovation and Transformation Centre, Federation University Australia, Churchill, Victoria, Australia; c Charles Sturt University, Port Macquarie, New South Wales, Australia; d Health Innovation and Transformation Centre (HITC), Federation University Australia, Mount Helen, Victoria, Australia

**Keywords:** Suicide prevention, Gambling, Commercial determinants of health, Social epidemiology, Public health, Commercial determinants of suicide

## Abstract

This paper presents a model of the commercial determinants of health in the context of gambling-related harm, suicidality and suicide. It outlines the ways the gambling ecosystem undermines suicide prevention efforts by driving harmful engagement with gambling. Using the dominant, orthodox discourse of ‘responsible gambling’, the ecosystem relies on the effects of addiction to underpin, sustain, and grow its power. Attempts to introduce effective interventions to prevent gambling-related harms are often blocked by the gambling ecosystem actors, using an evidence base that is biased by its focus on individual level causation and the attendant ‘responsible gambling’ responses. This emphasis on individual responsibility diverts attention from the practices of the industry, generates stigma and shame for those harmed, downplays serious harms caused by gambling, and contributes to the suicide toll. As most gambling activity is unrecorded, and systems for monitoring harms are underdeveloped, the true extent of these consequences have been largely invisible. This makes it more difficult to hold governments to account to regulate and prevent gambling-related harms, including suicidality and suicide. With growing evidence of harms linked to gambling, including suicide and increasing public concern, we present measures that could be adopted to disrupt these determinants and improve accountability to prevent harms and save lives.

**Research in context**

**Evidence before this study**

Gambling is recognised as a contributing or causal factor in suicide deaths. It has recently been described for the first time in Suicide Prevention Strategies in England (2023) and Australia (2025) as a driver or risk factor for suicide. Commercial aspects of the gambling system have been paid little attention due to the narrow focus on biomedical, individualised approaches to research and policy favoured by the ‘*responsible gambling*’ discourse. Suicide prevention efforts could be accelerated by taking greater account of the social and commercial determinants of health.

**Added value of this study**

We provide a model of the commercial determinants of gambling-related harm, suicidality and suicide. In doing so we outline pathways, practices, and systems that undermine suicide prevention efforts, and present countermeasures to address these. While taking an international perspective, examples provided here draw heavily on observations from Australia, which has a long history of legal gambling and is the largest per capita gambling market globally. Australia provides a cautionary example of how relatively unrestrained regulation can erode democratic systems and undermine population health. We show how the ecosystem has exerted power over governments to block reforms that would threaten their revenue streams. This results in a situation where a person experiencing a gambling-related suicidal crisis has limited or inappropriate tools or support.

**Implications of all the available evidence**

New streams of research and action are needed to inform government decision makers and enable them to protect health and prevent avoidable deaths. This includes identifying gambling in existing suicide monitoring systems, providing people who gamble with tools to control their losses, using centralised data systems to hold companies to account, and ensuring policy, research, education, and treatment are independent, and not influenced by vested interests.

## The growth of gambling-related harm, suicidality and suicide

Gambling harms extend beyond financial distress. They can include relationship breakdown, family violence, social isolation, mental and physical ill-health, and suicide.^1^ Harm affects not only the people who gamble, but their families, friends and communities.^2^ The legacy of harm can endure beyond crisis events, and harms can transmit between generations.^3^ Gambling disproportionately affects the most socially and economically disadvantaged,^4^, ^5^, ^6^ exacerbating and entrenching inequities.

While gambling can also cause harm at subclinical levels, gambling disorder is classified as a behavioural addiction, categorised alongside substance use disorders in the DSM-5-TR^7^ and ICD-11.^8^ Key symptoms of the condition include an inability to control gambling losses, and escalation of spending despite negative consequences.^8^^,^^9^ There is evidence of increased suicide mortality amongst those diagnosed with a gambling disorder^10^^,^^11^ and those who gamble.^12^ While estimates likely vary in part due to death investigation protocols, a substantial proportion of suicide deaths have been identified as gambling-related in Victoria, Australia (4.2%),^4^ and Hong Kong (19.4%).^4^^,^^13^

## The social and commercial determinants of health

As with gambling studies, the field of suicide prevention has primarily framed suicide as a mental health issue, emphasising individuals as key drivers of prevention.^14^, ^15^, ^16^ A public health approach is recognised as the appropriate response to addressing gambling harms^1^ and suicide^15^^,^^17^ at the population level. The growing field of the commercial determinants of health (CDoH) examines the ‘systems, practices and pathways through which commercial actors influence health and equity.’^18^ This concept supports a more expansive understanding of causality, and points not only to the industries that produce these products (i.e., enabling exposure to products designed with increasing intensity), but also the institutions or entities that benefit from revenues linked to these products. This suggests that commercial determinants are not only materialised as particular corporations, products and practices, but also (and importantly) as elements of human interaction with these. Recent work has argued that the suicide prevention field should consider the social^19^^,^^20^ and commercial determinants of health^21^, ^22^, ^23^ to move beyond individual or mental illness framings to a comprehensive public health approach. Here we describe how commercial forces may mediate or moderate engagement with gambling and how this may lead to suicidality and suicide.

**Fig. 1:**
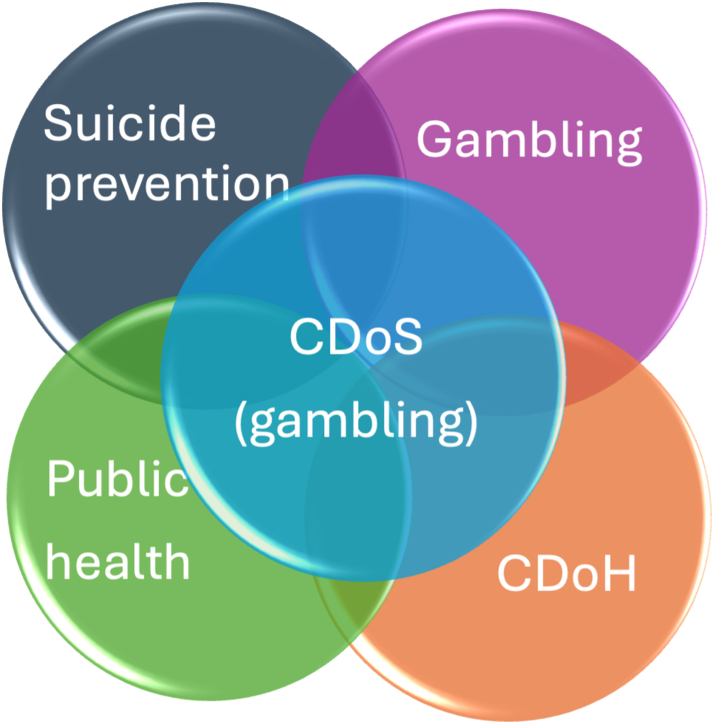
The commercial determinants of gambling-related harm, suicidality and suicide brings together four intersecting fields.

The CDoH have recently been extended to the conceptualisation of the commercial determinants of suicide.^21^ In this paper we adapt the conceptualisation of commercial determinants of suicide described by van Schalkwyk et al. (2023),^21^ to specifically examine gambling-related harm, suicidality, and suicide. In doing so we describe the intersection of suicide prevention, gambling, CDoH, and public health fields (Fig. 1).

## Suicide as an ‘embodied’ response to serious gambling harm

**Fig. 2:**
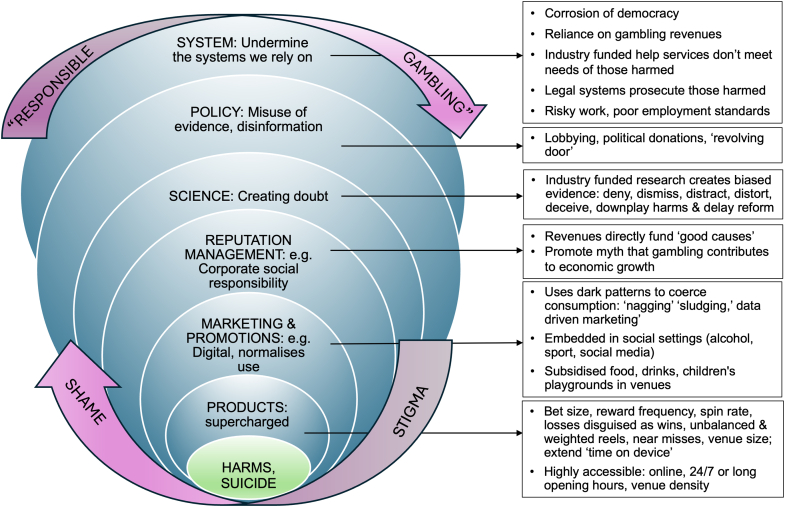
This model of the commercial determinants of gambling-related harm, suicidality and suicide and illustrates six layers comprising levels, practices and pathways through which the gambling ecosystem operates and undermines suicide prevention efforts. The ecosystem relies upon the orthodox discourse of ‘responsible gambling’ which generates stigma and shame for those harmed.

To outline our conceptualisation, we provide a model of gambling-related harms leading to suicidality and suicide which are the outcomes shown at the core of Fig. 2. The six layers surrounding the central core are an adaptation of the conceptual model of the commercial determinants of suicide developed by van Schalkwyk et al.^21^ Here, to avoid confusion with epidemiological levels (e.g., gene, individual, family, community), we describe *layers* which comprise of levels (product), pathways (system, policy), and practices (marketing, reputation management, science) that influence the production of harmful outcomes. In this model, the layers are a feature of the discourse of ‘responsible gambling’, which generate stigma and shame contributing to suicidality and suicide. This model is provided to generate discussion and is intended to be dynamic, rather than definitive. The degree to which the gambling ecosystem succeeds in deploying its power at each layer in the model may explain the distribution and extent of gambling-related suicide in populations. Fig. 2 depicts the layers separately, however, we acknowledge that in practice these may overlap or interconnect.^21^ Further, while in some contexts the effects of elements within each layer may be cumulative, not all elements are necessary in all contexts to generate severe harms. Some elements may circumvent layers in different contexts. As Krieger writes on causal logic, ‘events at one level can directly and profoundly affect nonadjacent levels, instantly and persistently, without intermediaries.’^24^

## What drives the commercial determinants of gambling-related suicide?

**Fig. 3:**
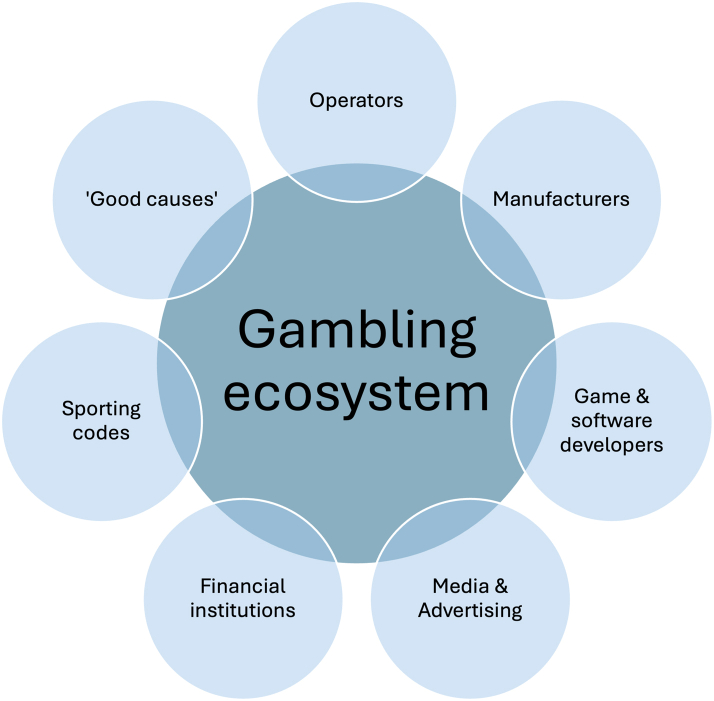
The ‘gambling ecosystem’ refers to a range of commercial actors that derive financial benefits from the growth of gambling.

The commercial entities that form the gambling ecosystem are actors that benefit commercially from the continued growth of this industry. They may act individually or collectively to block or erode reforms that jeopardise revenue streams. The ecosystem includes gambling operators, manufacturers, game and software developers (including associated peak lobby groups), advertising, media (including social media platforms and broadcasters), financial institutions (including banks and payday lenders), charities, and sporting codes (Fig. 3).

Their influence can be constrained or enabled by governments that are expected to regulate in the public interest. Industries that produce addictive products differ from other categories of damaging industries. Ecosystem entities often operate across other unhealthy commodities, like alcohol,^25^ highlighting the value of a coordinated approach to disrupting determinants across industries and products, and thus avoid research or policy ‘silos.’^26^ The ultra-processed food industry has been described as ‘behaviourally challenging’ or, in the case of fossil fuel industries, ‘contextually damaging.’^27^ Notably, because of its dependence on addicted users, the gambling ecosystem generates an ‘addiction surplus’–that is, financial returns after costs are produced at levels in excess of non-addictive commodities.^28^ This excessive consumption of addicted consumers maintains and expands the power of this ecosystem, using strategies outlined in Fig. 2. These arrangements produce the normalised social world in which products are licensed, operated, and consumed.

The six layers depicted in Fig. 2 demonstrate how various aspects of commercial activities contribute to the production of gambling-related harm, suicidality, and suicide. These may accumulate to increase financial returns, but also to increase the likelihood of adverse consequences for those who use these products. We contend that effective interventions to disrupt the commercial determinants of gambling-related harm, suicidality and suicide will be developed only by disrupting the practices, institutions, and systems that sustain the commercial determinants of health and suicide.

## Increasing intensity, availability, marketing and promotion of gambling products

**Fig. 4:**
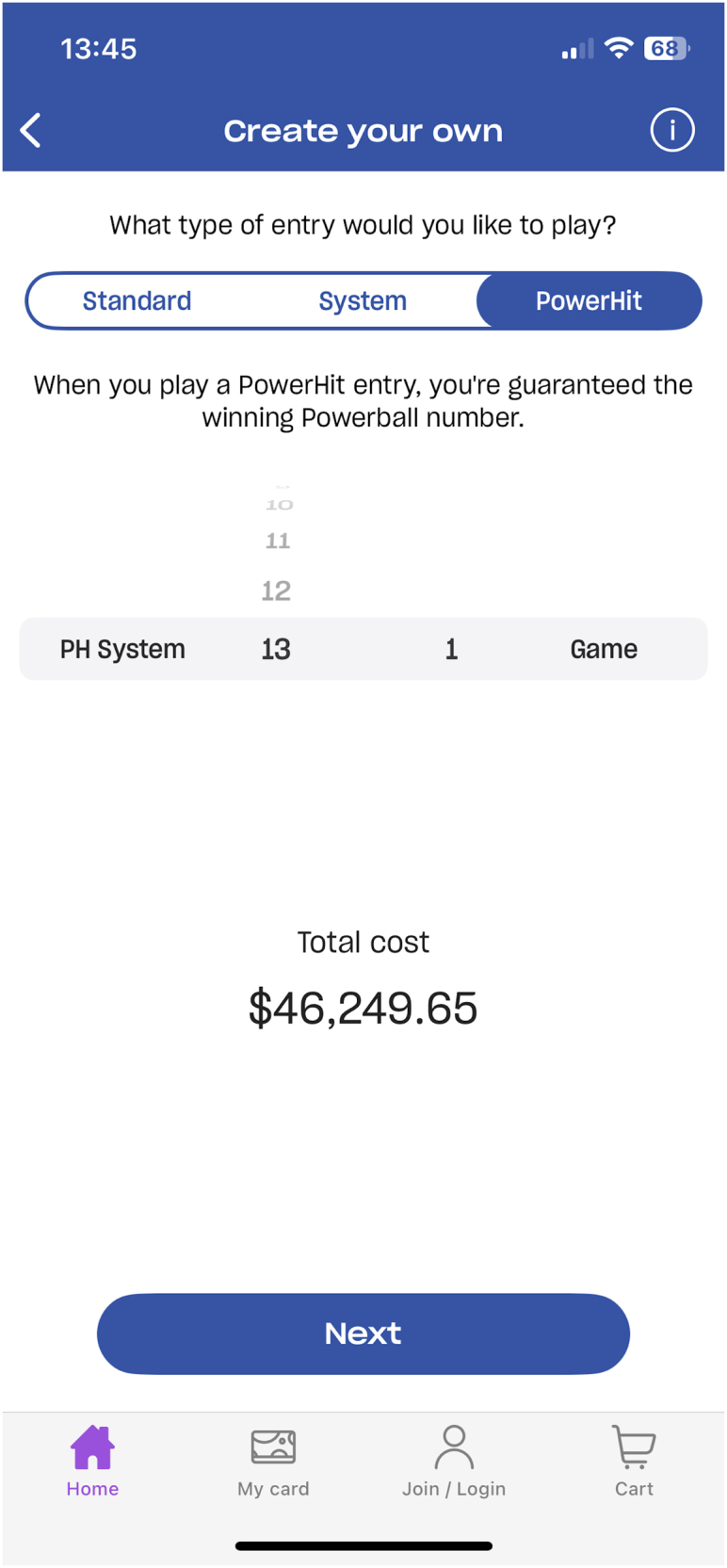
Cost of a maximum Powerball ticket.

Gambling products present distinct harm profiles, many of which have intensified over time.^29^ Schull reported that gambling industry representatives describe their aim is to maximise revenue per available customer (revpac), and encourage ‘playing to extinction’, the point at which a customer has exhausted all available funds.^30^ Given the evidence of links between gambling and suicide, and in the context of our model, this phrase acquires even more significance. Physical gambling products (such as electronic gambling machines [EGMs]) have evolved from mechanical reels into fully digitised devices incorporating features designed to encourage extended use and expenditure.^31^ Digitisation has also accelerated the intensity of gambling. It permits features in games that increase complexity, speed of operation, cost of wagers, and volatility, and provide ‘frictionless’ transactions; all features that can undermine executive function.^32^ Gambling products are now commonly delivered via mobile applications allowing operators to provide–and promote–their products to people almost anywhere, at any time.^1^ Products that were once regarded as relatively benign, such as lotteries, now allow for substantial spending. Lotteries are also drawn more frequently, increasing the capacity for more substantial losses. Subscription features that enable a customer to ‘set and forget’ ticket purchases.^33^ A single ticket in the Australian lottery game ‘Powerball’ can cost as much as AUD$46,249.65^e^ (Fig. 4).

Although data is not publicly available to determine how often high-cost tickets are purchased, it is unlikely many would spend this much on a single ticket. The key function of such a high-cost ticket is to subtly encourage people to increase their spending; purchasing a ticket for a hundred or even several thousand dollars may begin to seem reasonable in comparison.

Digitisation enables consumer data to be harvested to target promotions that encourage consumption, such as persistent promotional messaging (‘nagging’) and ‘dark nudges’^34^ such as promoting increased spending (Fig. 4), or particular products highly unlikely to return money. One example of this is the ‘mulitbet’ wager which relies on a sequence of outcomes occurring, they have a lower chance of return than single outcome events. Companies use algorithms to hyper-target advertising that both harvests and responds to data collected through customers unique online activity to direct their behaviour.^35^ Behavioural science strategies using online choice architecture^f^ encourage people to continue spending, despite an intention or preference to stop. For instance, sludging^36^–the opposite of a health promoting ‘nudge’–is a tactic used in this context to make withdrawing funds or closing online accounts difficult for customers,^34^ encouraging continued consumption. Digitisation could be used for harm prevention purposes, but the slow adoption of these systems in physical venues might also be conceived as a form of sludging. In Australia, universal account systems that allow people to set loss limits have been strongly resisted for the past 15 years, despite a key government agency, the Productivity Commission, recommending adoption in 2010 (see Panel 1).^44^ Instead, gambling operators make it difficult for their ‘best’ customers to stop, for instance by relying on manual, paper-based self-exclusion.^48^ This requires people who wish to self-exclude to complete paper forms for each venue from which they wish to exclude, and provide a passport photo which is meant to enable venue staff to recognise the excluded individual and intervene if they attend the venue.

Panel 1 **How ClubsNSW exerts power over government.**

**NSW clubs form the largest gambling network in the world**

NSW was the first state in Australia to introduce EGMs in 1956. In 2024 there were 87,632 club and hotel based (non-casino) EGMs operating in NSW.^37^ Three quarters of these machines are in local club venues, which, as not-for-profit entities, receive preferential tax concessions compared to hotels. Some clubs are very large; seventy-four club venues (8%) operate 200 or more EGMs. The socioeconomically disadvantaged suburbs of western Sydney are home to the largest club venues, with four of the state’s most five profitable venues in these local government areas. Evidence shows that venues operating EGMs generate economic rent from the poorest members of the community.^1^^,^^5^^,^^38^ The Bankstown Sports Club is the largest club in NSW, operating 725 EGMs in one venue. In the 2024 financial year, the Club earned AUD$105 million in ‘gaming’ (EGM) revenue–or losses derived from their members. While gambling revenues comprised 70% of 2024 earnings, the word ‘gambling’ does not appear in the Clubs annual report.^39^

**Club grants to ‘good causes’**

The NSW government requires clubs generating gambling profits over AUD$1 million to allocate 0.4% of profits to the ClubGRANTS scheme. Clubs can apply for a 1.85% tax rebate on EGM profits over AUD$1 million. Community groups can apply for grants in their local area and applications are usually assessed directly by clubs granting funds, i.e., without a government intermediary. The ClubGRANTS guidelines state these grants are “… designed to ensure that larger registered clubs in NSW contribute to the provision of frontline services to their local communities; and to ensure that the disadvantaged in the community are better positioned to benefit from the substantial contributions made by those clubs.”^40^ The share of NSW government taxes contributed by clubs is lower (40.3%) than hotels (55.2%) despite clubs venues collectively making a larger proportion of EGM profit.

**Lobbying prevented urgent reforms**

ClubsNSW is a powerful lobby group that represents NSW EGM club operators. In 2010, 2014 and 2018 NSW Premiers signed memoranda of understanding with the lobby group in advance of each state election, to promise to maintain tax rates and existing gambling operating arrangements. No other economic sector regularly receives such guarantees in NSW. These memoranda declare a ‘commitment to a gambling policy environment informed by research and evidence and a strong commitment to harm minimisation’. However, interventions to support this goal are favourable to maintaining profit streams, including maintaining existing tax and grant schemes, ‘jackpot forfeitures for excluded gamblers’ and ‘**voluntary venue-based** pre-commitment’^41^ (emphasis added to distinguish this from **universal jurisdiction wide** pre-commitment recommended by the Productivity Commission shown to be effective in other countries).^42^ In January 2011, it was reported that ClubsNSW executives were trained by the National Rifle Association in the USA in community based political campaigning.^43^ This occurred during the most significant attempt at gambling reform in Australia (2010–2012) when the Australian Government attempted to legislate key recommendations of the Productivity Commission 2010 inquiry, including universal precommitment on EGMs.^44^ ClubsNSW led a campaign that threatened politicians in marginal seats, and ultimately succeeded in blocking the reform.^45^ Following a series of inquiries into Australian casinos commencing in 2019^46^^,^^47^ some casino venues have now been required to introduce universal precommitment systems on EGMs, largely in response to evidence of widespread money laundering. Fifteen years after the Productivity Commission recommendation, no Australian government has succeeded in implementing universal precommitment statewide.

Direct advertising of some forms of gambling, such as EGMs and casinos, is prohibited in Australia. However, operators use indirect promotions such as free or subsidised food and drinks and provide children’s play areas (e.g., slides, ball pits, video games) and ‘kids eat free’ offers to encourage families to attend venues.^38^ Advertising of gambling through sport is widely regarded as relentless in Australia, despite the introduction of minor restrictions such as industry codes banning the broadcast of advertisements on mass media during the watershed hours of children’s television viewing hours.^g^ Broadcasters, sporting codes, and sporting identities can generate substantial revenues through this advertising. The broader gambling ecosystem has used its power to block a range of measures to reduce harm from online gambling, including avoiding advertising prohibitions unanimously recommended by an all-party parliamentary enquiry in Australia in 2023.^49^

By promoting gambling as fun, entertaining or glamourous, advertising compromises understanding of the severity of possible harms.^1^ Those experiencing harm are accordingly often isolated, and likely to believe themselves at serious fault. This contributes to stigma, can prevent help-seeking, and may exacerbate feelings of guilt and shame,^50^, ^51^, ^52^ which is a known mechanism in the relationship between gambling and suicide.^52^

## Managing the reputation of the gambling ecosystem

Linking gambling industry funding to worthy or charitable causes has been described as creating an ‘alibi’ to legitimise gambling operations.^53^ In many parts of the world, governments historically organised lotteries to fund social and good causes. In several European countries, gambling companies–often publicly owned–fund a wide range of activities including substance use treatment programs, sports, and culture.^54^, ^55^, ^56^ In Spain people with visual impairment or other different abilities are employed by the lottery operator ostensibly to reduce unemployment among this population.^56^ In Western Australia, LotteryWest directly funds the Health Promotion Agency, Healthway. The Hong Kong Jockey Club is described as one of the world’s leading charitable donors. Since 2002 it has funded the Hong Kong Jockey Club Centre for Suicide Prevention Research,^49^ and in 2023 established a Global Health Initiative, comprising a partnership between Hong Kong University, the International Vaccine Initiative, and Cambridge University.^57^ Part of that initiative includes establishing a perpetual Hong Kong Jockey Club Professorship of Global Health Cambridge University, via a gift of GBP4.2 million.^58^

In Victoria, Australia, hotels pay an additional 8.33% tax on their EGM revenues, compared to social clubs. The funds from this extra tax are allocated to the *Community* Support Fund. Funds are held in a trust administered by the Department of Treasury and Finance. Annual financial statements are publicly available and provide a brief overview of organisations and projects funded. These include a range of community and government activities. Some grants, although not all, directly relate to gambling, such as AUD$37 million to the Department of Justice and Community Safety for “addressing gambling harm and applying a contemporary approach to gambling regulation,” AUD$1.5 million for Regional Food Relief Hubs to “receive, sort and store larger quantities of rescued and donated food, for distribution to frontline community organisations” and AUD$10,000 for the Victorian Aboriginal Funeral Service “… to support the replacement of a damaged hearse …” A different scheme, the Community Benefits Scheme, allows social clubs to *avoid* this extra tax if they demonstrate that they have contributed at least 8.33% of their EGM revenue to good causes. However, because clubs can claim their own operating costs as charitable contributions, in practice this means that average contributions amount to much less. A 2019 study found that an average of 1.5% of such claims were actually philanthropic or charitable, whereas 82% of allowable claims were operating costs including wages.^59^

The governance arrangements for the Victorian Community Support Fund differ from those of the New South Wales (NSW) ClubGRANTS (see Panel 1) scheme, and with the Victorian Community Benefits Scheme, in that the government acts as an intermediary in the administration of the former.

Beneficiaries of these arrangements can develop dependence on these revenues. Kingma argued in the context of Dutch gambling policy “… at first the lottery was primarily dependent on the good cause and then, gradually, the good cause became increasingly dependent on the lottery.”^53^ This symbiotic, reflexive relationship creates dependencies in both directions, with the ‘good cause’ also providing cover for the gambling entity to avoid reforms that could threaten their revenue streams (see Panel 1).

The influence of gambling industry actors is one step removed in the Victorian Community Support Fund, but it remains problematic. Adams argues that these direct links carry a chain of influence that allow industry to be seen as addressing harm, and which may fund activities that divert attention from more effective measures that might reduce harms, and by definition, reduce profits.^60^

A major review of Australia’s taxation system in 2010 noted the not-for-profit arrangement for clubs was ineffective, and recommended that governments eliminate these tax concessions.^61^ However, no changes have yet been made. Such taxation arrangements expand the gambling ecosystem by linking gambling revenues to community groups providing sporting, cultural, social care, and support services. This creates dependencies on gambling revenues and procures a form of social license. It also serves as a barrier to reform by creating resistance to measures that may threaten these revenues.^62^

## Distorting scientific evidence

Conflicts of interest influence the evidence base in a range of areas relevant to public health.^63^, ^64^, ^65^, ^66^ The gambling studies field is notable amongst these.^67^^,^^68^ Influence operates across the research cycle via direct funding of ‘safe’ research, through framing of research agendas and questions, identification of study populations, methods used, and the communication of findings.^69^ This includes activities such as hosting or sponsoring scientific conferences,^70^ spaces known for ‘grooming’ researchers and creating ‘moral jeopardy.’^27^ Conflicts of interest not only create opportunity costs as ‘safe’ research diverts resources from more urgent research likely to produce knowledge that could lead to more effective interventions,^71^ but can also mislead decision makers and the public who rely upon this evidence to respond to harms, for instance by downplaying harms linked to addictive products, or complicating causality.

A significant body of research in gambling studies has focussed on ‘comorbidities’ associated with ‘problem’ or ‘pathological gambling.’ While this has merit, for instance to assess priorities in treatment, the volume of this research in gambling studies is arguably outsized. Further, such analysis frequently overlooks relevant factors, such as more readily modifiable changes in population exposure to high intensity forms of gambling, such as EGMs. For instance, the influential ‘pathways model’ asserts three gambler sub-types; behaviourally conditioned, emotional, and impulsive ‘problem gamblers.’ This third group are said to

… *demonstrate elevated levels of impulsivity that is highly correlated with measures of psychopathology and clinical criteria for antisocial personality disorder. These gamblers exhibit a family history of problem gambling, early onset, more severe levels of gambling, a history of suicidal ideation and/or attempts, co-morbid substance dependency, antisocial and narcissistic traits, affective instability, widespread dysfunction in non-gambling related areas and unresponsiveness to treatment*. –^72^^p. 97^

The implication of this is that gambling harms are an artefact of other, individual conditions, such as antisocial personality disorder. An overemphasis on comorbidities distracts from the potential to modify the arrangements under which gambling is provided, which would provide greater protections to segments of the population who are likely at elevated risk of harm.

The first study of gambling-related suicide in Australia was published in 1998. The authors reviewed 44 suicide cases between 1990 and 1997 where the Victorian coroner had made a ‘putative’ gambling finding, reporting an “inexplicable peak in suicide deaths in 1996”, which they reported doubled from 7 to 14 cases between 1995 and 1996 then reduced to 8 in 1997.^73^ This trend, however, is far from inexplicable. Firstly, gambling-related suicide cases likely increased over this time because of a major change in availability of the most harmful form of gambling. EGMs were introduced into Victorian community hotels and clubs during 1992–93, increasing in number up to the present cap of 29,372. The state’s only casino opened in Melbourne in November 1993 with a license to operate 2750 EGMs. Both events led to a substantial increase in gambling losses: AUD$1.4 billion in 1993–4, the first year for which this data is available, to AUD$2.7 billion in 1996.^74^ EGMs are responsible for over half the gambling problems in Australia,^75^ are the most common reason for people with a gambling problem to present to mental health crisis services in Melbourne,^76^ and the gambling product most likely to be associated with suicide.^4^ Secondly, finalisation of coronial findings can lag by several years. Given the 1998 publication date, 1997 cases would likely have still been open (i.e., not concluded) at the time of publication. Neither of these explanations are acknowledged.

A subsequent protocol for ‘psychological autopsy’ to identify gambling-related suicides takes a narrow case definition, instructing, with no reference to any evidence, that:

*… it is necessary to exclude cases where there is evidence of a chronic history of psychiatric illness …, interpersonal problems characterized by a chronic history of social isolation and withdrawal from early childhood, evidence of childhood history of sexual or physical abuse, and chronic substance abuse … While these factors may lead a person to seek emotional escape in gambling behavior, they are capable of accounting for suicidal urges in their own right. … where a co-morbid condition is present, it cannot be concluded with any degree of certainty whether the gambling, the co-morbid condition, or an interaction of the two were instrumental in causing the suicidal act*^77^^p. 12–13^

Gambling can also be the dominant factor in a suicide death, but this consideration was not offered in this article. The lead author now discloses extensive gambling operator funding (for example^78^), although at the time of the article no such disclosures were forthcoming. Associations include being a longstanding consultant to Crown Casino on ‘responsible gambling’ practice^46^ up to the time that Crown Casino was the subject of a Royal Commission, the findings of which including pointed commentary on the failure of its ‘responsible gambling’ program.^46^ We do not suggest that these authors have acted in bad faith. However, receipt of funding from vested interests may readily give rise to the appearance of conflict of interest, and may have an unintended effect on issues such as the selection and interpretation of data, and the selection of research questions. One of these authors also publicly cast doubt on the potential effectiveness of precommitment during the negotiation of relevant federal legislation in 2011, asserting on the Australian Broadcasting Commission (ABC) news program 4 Corners:

*I don't believe that pre-commitment is going to have its desired effects. I think there may in fact be an unintended consequence where gamblers set higher limits and then will gamble more amounts of money to meet those particular limits and that's a detrimental effect and may aggravate the problem for a proportion of gamblers. Secondly, I think the cost benefit needs to be re-analysed. I think the cost of implementing the system is not going to be worth the benefit it does achieve and much of that money could in fact be spent on more effective responsible gambling measures.*^79^

A subsequent review references ‘evidence’ of unintended effects. However examination of these references shows no support for this conclusion. In fact, the authors of this paper found:

*The presence of methodological limitations related to small unrepresentative samples … preclude any conclusive statement on the effectiveness of precommitment systems on gamblers*^80^^p. 227.^

This body of work downplays the role of gambling in suicide, and undermined confidence in the likely effectiveness of an important gambling harm prevention tool. Discussions of data, protocols, and unsubstantiated claims of ineffectiveness support industry narratives, and undermine possible progress towards effective harm prevention.

## Undermining the systems we rely on

Donations from gambling ecosystem actors to politicians are underestimated due to gaps in disclosure laws. Despite underreporting, donations from the gambling industry are substantial.^25^^,^^81^ The common phenomenon of ‘the revolving door’ involving parliamentary staff and parliamentarians, hired by the gambling ecosystem on retirement from political careers, provides privileged access to inside information.^81^^,^^82^ This is one possible reason for politicians to delay meaningful actions that could address gambling harms.

Evidence shows that operators often engage in predatory practices to encourage people to continue gambling against their better interests, in contravention of responsible gambling codes of conduct.^83^ The Victorian Royal Commission found “Crown Melbourne’s conduct is of the most egregious kind and it involved systemic and repeated failings in relation to a wide range of activities”^46^ ^p. 64^ citing money laundering connected to organised crime and underpayment of taxes among these. It also found that:

*Crown Melbourne had for years held itself out as having a world’s best approach to problem gambling. Nothing can be further from the truth. The Commission heard many distressing stories from people whose lives were ruined by gambling but whose situation might have been improved if casino staff had carried out their obligations under Crown Melbourne’s Gambling Code.*^46^^- p. 3^

Unfortunately, such conduct is not limited to casinos. A study of suburban gambling venues revealed similar patterns.^38^ Despite a series of recent Royal Commissions into casino operations in Australia uncovering substantial, extensive and entrenched illegal activity, including money laundering, no gambling executive or board member has been charged with criminal offences.

Evidence demonstrates that drug courts can reduce drug use, prevent recidivism, and reduce costs for people using substances.^84^ However, similar programs for people experiencing gambling addiction are almost non-existent.^85^^,^^86^ Yet the justice system, perversely, penalises those harmed by gambling; a substantial proportion (e.g., 18%)^87^ of some prison populations consist of people who experience gambling harm.^88^ An inquiry into money laundering in NSW hotels and clubs reported that people dealing drugs were gambling ‘on a large scale’ with the proceeds of that crime, indicating that a substantial volume of drug sales were supporting gambling addiction.^89^ Imprisonment and drug use are also factors that place people at heightened risk of suicide. Providing people with tools to limit their gambling could not only prevent money laundering, but also reduce major social harms such as the sale and use of illegal drugs, as well as suicides that may be more directly linked drug use and imprisonment. The inequitable treatment of those overseeing gambling operations, compared to those harmed by gambling, may compound frustration, stigma, and shame, accelerating risks of suicidality and suicide.^50^

## Actions to disrupt the commercial determinants of gambling-related harm, suicidality and suicide

Effective population-level measures to prevent and reduce harm from gambling have been identified^90^^,^^91^ and implemented in some countries. Norway^92^^,^^93^ and, to a lesser extent, Finland,^94^ are among those that have introduced effective tools such as universal account systems to allow people to limit gambling losses. Notably, both countries have had substantial publicly owned government monopolies, although Finland’s monopoly is due to end in 2027. Centralised universal account registration provides a critical mechanism for people who gamble to set loss limits, one important tool to control spending. Data vaults are available in several European and South American countries,^95^ and have been used to evaluate gambling operator compliance with laws and track emerging trends and indicators of harm, enabling rapid responses to address non-compliance or adverse events.

A range of measures are available to disrupt the pathways, practices, and systems of power that undermine suicide prevention efforts. Table 1 summarises how the six layers in Fig. 2 operate and suggests countermeasures to disrupt the commercial determinants of gambling-related harm, suicidality, and suicide.

**Table 1:** Summary of ecosystem influence and countermeasures.

Layers	Practices and pathways	Objectives	Effects	Countermeasures
Products	• Intensification of reward and volatility • high accessibility (opening hours, 24/7 online, density of venues)	• Increase time on device • Maximise ‘revpac’ • Encourage ‘playing to extinction’ (where customer exhausts all available funds)	• Increased spending increases operator and ecosystem profits • More intense and addictive products generate financial surpluses that can support corporate political activity to maintain/expand business and resist regulation that might reduce profits.	• Require centralised universal account registration for use of gambling products • Improve product safety and strengthen laws and regulation of gambling products • Provide tools to limit losses (money, time) and self-exclude • Ban features and characteristics that mislead users • Resource regulators to ensure they understand products they are regulating • Establish post-marketing surveillance of harms associated with gambling products
Advertising and marketing	• Social, mass media advertising campaigns • Sponsorship of sports and culture, signage • Targeted advertising, promotions direct to consumer • Online choice architecture: including sludging, nagging	• Increase engagement with gambling • Prevent defection of ‘best’ customers from gambling • Encourage ‘playing to extinction’	• Normalise gambling, including for children and young people • Increase market share • Spending on more productive or health promoting activity is diverted to gambling	• Ban gambling advertising and sponsorship • Require gambling businesses to seek approval to engage in advertising promotion campaigns with regulators and health authorities during phase out period. • Regulate digital marketing practices and use of algorithms that target consumers
Reputation management	• Funding sports, culture, social projects and other ‘worthy causes’	• Enhance reputation of gambling operators as caring entities who provide vital financial support	• Perceived dependence on gambling revenues, despite significant costs • Create a social license for gambling operations • Legitimise status quo: ‘business as usual’ prevails • Reduce support for meaningful reforms that could compromise beneficiaries	• Remove not-for-profit status of gambling operators for the purposes of research and funding good causes • Revise taxation of gambling products to decouple direct funding of good causes, funding these instead through consolidated revenues • Decouple direct funding of gambling revenues to good causes and other community activities: revise tax arrangements to fund good causes from consolidated revenues • Capture negative externalities, recognise the ‘cannibalisation’ of non-gambling businesses by those that can afford to subsidise other elements of their business
Science	• Influence at all elements of the research cycle, including framing of research agendas and questions, funding, reporting, discredit unfavourable research/ers;	• Avoid adoption of meaningful reforms that would reduce revenue streams to the gambling ecosystem: Distract, deny, deceive, distort, delay, dismiss	• Distortion of the evidence-base upon which policy is made, meaningful reforms delayed, diluted or abandoned	• Measure and report the true health, social, economic cost of gambling by capturing and improving screening, diagnosis, treatment monitoring and surveillance of key indicator data (e.g., flag gambling in suicide registers, ED attendances, GD diagnosis, treatment, debt, crime data) to inform prevention and enable communities to hold governments to account. • Funders, editors, publishers, researchers, ethics committees work to improve accuracy and scrutiny in reporting of conflicts of interest • Universities policies prohibit gambling industry funding of researchers and their institutions • Increase the quantum of funds available for independent research to prevent gambling harms, such as through regulatory settlements
Policy	• Gambling ecosystem donations to political representatives • Lobbying politicians and staffers • Revolving door between politics and gambling ecosystem	• Purchase favourable policy from decision makers • Block reforms that could reduce profits • Capitalise on ‘insider’ knowledge by advising or taking up employment with gambling ecosystem entities post-politics	• Expand/maintain industry revenue streams • Corrosion of democratic processes and democracy	• Develop, resource, implement and evaluate national gambling control strategies and plans • Ban political donations from gambling and other addictive consumption and health- harming industries (some operate in more than one industry). • Prohibit revolving door practices (e.g., ecosystem actors cannot recruit parliamentarians or parliamentary staff for at least 3 years upon leaving parliament) • Provide live donations and lobby register data, publish politicians diaries • Require submissions to inquiries to disclose funding and other conflicts of interest • Support independent media to examine and expose links between industry and undue influence over government • Explicitly prioritise population health in legislation, regulation and policy • Prioritise suicide prevention in gambling policy and regulation
System	• Visible, direct links between gambling revenues (taxation and also grants provide tax benefits) good causes, treatment and support	• Maintain the myth that gambling only harms a small minority; there are major economic benefits to governments and communities • Reduce community and government support for effective reform measures	• Harms are under recognised and responses under resourced • Support provided to those harmed is often inadequate, inappropriate and stigmatising	• Establish centralised data vaults of gambling accounts and provide access to police, coroners, regulators and researchers to examine trends and design responses to reduce harms and hold operators to account • Increase penalties and prosecute operators who breach the law • Establish monitoring and surveillance systems that measure the true extent of harm caused • Resource death investigators to routinely and systematically capture gambling and code consistently in mortality datasets. • Reduce dependence on gambling tax revenues by channelling these through consolidated revenues and ensure these are independently administered • Capture negative externalities through increased taxes on gambling ecosystem entities • Increase penalties for entities that breach the law and regulatory settlement funds to resource efforts to address harms • Require the legal system to formally recognise the addictive nature of gambling products and gambling disorder and addiction. Reflect this in sentencing by instituting diversion programs

### Establishing monitoring and surveillance systems for gambling

The dominance of the responsible gambling discourse, which promotes gambling as ‘fun’, and generates stigma and shame for those harmed (Fig. 2) has undermined efforts to build adequate public health systems to monitor health harms. Systems to identify the true extent of harms caused by gambling need to be improved, using the example of monitoring and surveillance systems established for alcohol and drugs.^1^ Suicide rates are one key health indicator of the extent of gambling harms. Under existing arrangements, knowledge that gambling might be linked to suicide is limited. Death investigation protocols do not require routine consideration of gambling in cases of suicide, meaning a gambling-related suicide may only be identified if evidence emerges spontaneously in the death investigation process. This underlines the need for other ways to readily identify people who gamble in the community.

Gambling is a behavioural addiction, where no substance is physically ingested.^8^ Unlike alcohol and drugs, there are currently no biomarkers to identify gambling use or ‘toxicity’. Evidence of the involvement of gambling may however be indicated in:

• Financial records, albeit that some may only indicate debts, without identifying the source of the debts;
• Health records, although few health professionals routinely screen for gambling problems; and gambling disorder is rarely diagnosed; and/or
• A suicide note left by the deceased that discusses gambling harms, which are typically only left in a small proportion of cases.^96^

Measures to improve prevention, treatment, operator and government accountability (universal account registration, centralised storage of accounts in data vaults, in addition to improved prevention tools, screening and diagnosis of gambling disorder by clinicians) would also assist in identifying people who gamble in the community for the purpose of death investigation.

Transparency in gambling provision and usage, including via universal accounts,^97^ is possible and important. Gambling operators and regulators use gambling sales or transaction data for commercial or taxation purposes. User-level data contains valuable insights into the frequency of engagement, volume of losses, and patterns of product use. These data should be provided to consumers to inform them of spending and could be investigated by regulators to prevent harm and ensure operator compliance with duty of care obligations. De-identified data should also be available for independent researchers to reduce costs and improve the quality of research that seeks to prevent and reduce harm from gambling.^97^ Account data should be provided to death investigators to provide insight into the gambling behaviour of the deceased. This could support prosecution of gambling ecosystem actors who engage in predatory practices. If gambling activity is not traced, operators may avoid scrutiny for breaching their duty of care and avoid detection for money laundering. In growing recognition of the link between gambling and suicide, the British Gambling Commission now requires gambling operators to report deaths by suicide as a condition of their license from April 2024. The Commission has committed to releasing data on the number of reports they have received when they have at least one full year of data available, noting that gambling businesses may not be aware of these deaths. Such a system would be improved by instead allowing the regulator to cross-check gambling account information stored in a data vault with suicide registers.^98^

### Strengths and limitations of this model

Research on the CDoH has been critiqued for being conducted in product ‘silos,’ which can undermine the coherence of policy responses.^26^ Arguments have also been made that CDoH research is at a point where it must move beyond description to theory building,^99^^,^^100^ measurement, and action.^101^ While describing a conceptual model, we seek to contribute to this agenda by outlining countermeasures to support action alongside this model. However, these measures are not exhaustive and require extension and/or adaptation to local contexts. Local context is important, both within and between countries, not only in terms of the degree to which resources are available to respond, but also how gambling, and responses to this, may be shaped by values and norms. Religions and cultures influence the way gambling is regulated and provided, including the degree to which it is normalised, restricted, or even prohibited. For instance, Islamic teachings consider gambling to be *haram*, resulting in gambling being prohibited in predominantly Muslim countries like Indonesia, Türkiye, and Bangladesh. Utah is now the only mainland US state to ban gambling. In other contexts, gambling is provided through a government monopoly (Norway) or restricted to tourists (e.g., Singapore, North Korea). Some countermeasures we have outlined assume a context where gambling tax revenues are collected by governments and where gambling is legal. The interventions we have discussed are neither prescriptive nor completely comprehensive. Different situations require responses tailored to the cultural context.

## Conclusion

The data that governments do—or do not—collect and publish has important implications for accountability and population health.^102^ Studies that emphasise individual level factors or do not take sufficient account of social and commercial conditions can underestimate the scale of the problem, divert attention from factors that drive population health and inequities, and distort the evidence base. Downplaying serious harms linked to gambling products suits the objectives of the gambling ecosystem. It misleads decision makers and the public about the scale of harm, enabling them to avoid or delay reforms that may threaten revenue streams, further entrenching the power of the ecosystem. Ignoring systemic factors will undermine progress to prevent suicide. Further, adopting erroneous models or theories about specific activities (such as those encapsulated in the ‘responsible gambling’ discourse)^103^ may lead to significant unnecessary harm, including suicides. As Krieger notes:

*if we get our theories wrong, we can do great harm; if we get them right (or at least use better ones), we stand a better chance at generating valid knowledge relevant to explaining disease distributions and altering them for the good.*^102^^p. 292^

By describing for the first time the ways in which the commercial determinants of gambling-related harm, suicidality, and suicide operate, we bring the CDoH to suicide prevention. In doing so we take a more expansive view of how populations embody the political, economic and cultural contexts in which they live. Describing the commercial determinants of suicide can clarify causal logic and draw attention to responses that will be most effective in improving health, preventing avoidable deaths, and reducing inequities.^102^ This can also help to build monitoring and surveillance systems that provide data hold governments accountable by highlighting the need for urgent policy action. It can also help to inform theories that can better address the influences driving suicide.

## Contributors

AR: conceptualisation, literature search, figures, study design, data collection, data analysis, data interpretation, writing–original draft, writing–review and editing.

SM: supervision, writing–review and editing.

KS: writing–review and editing.

BK: supervision, writing–review and editing.

## Declaration of interests

AR holds a fellowship funded by Suicide Prevention Australia. In the past five years she has received funding from the Victoria Responsible Gambling Foundation, which was supported by allocations from the Community Support Fund, a government administered trust fund constituted from direct taxes on EGMs in hotels, the Winston Churchill Memorial Trust and ANROWS. She is a member of the WHO meeting on gambling and received consultancy funding from WHO. AR has also received honoraria to review grants by the British Academic Forum for the Study of Gambling, which is administered via Gambling Research Exchange Ontario, funded by regulatory settlements from gambling companies who have breached the law.

Others have nothing to declare.

## Footnotes

e This ticket is a 13 number ‘system’ ‘Powerhit’ entry in the Australian lottery product Powerball, drawn weekly.

f This refers to the design of the online environment which influences how people make decisions about how they spend their time and money.

g Advertisements can still be aired after 8:30 p.m. but are restricted to breaks. This restriction has largely been ineffective in preventing young people from being exposed to gambling advertising, given football or rugby matches often extend after 8:30 p.m. and young people under 18 years of age do not stop watching television because of arbitrary definitions of children’s viewing hours.
