# Corrigendum to ‘Playing to extinction’: the commercial determinants of gambling-related harm, suicidality and suicide, The Lancet Regional Health – Western Pacific Volume 63, October 2025, 101685

**DOI:** 10.1016/j.lanwpc.2026.101814

**Published:** 2026-02-09

**Authors:** Angela Rintoul, Suzanne McLaren, Kerrie Shandley, Britt Klein

**Affiliations:** a Centre for Mental Health and Community Wellbeing, University of Melbourne, Australia; b Health Innovation and Transformation Centre, Federation University Australia, Churchill, Victoria, Australia; c Charles Sturt University, Port Macquarie, New South Wales, Australia; d Health Innovation and Transformation Centre (HITC), Federation University Australia, Mount Helen, Victoria, Australia

The authors regret that in our original article we stated: “In Western Australia, LotteryWest directly funds the Health Promotion Agency, Healthway.” However we now understand this statement is factually incorrect. We have changed the article to better reflect the arrangements described below. The relevant section now reads:

“In Western Australia, the Health Promotion Agency, Healthway, and Lotterywest, are independent entities. However they are linked by an agreement, via which Healthway paid Lotterywest $3.7 million in 2024–25. Under this agreement, Lotterywest provides employment services, office accommodation, and IT services for Healthway. The CEO for Lotterywest is also CEO of Healthway.”

The authors would like to apologise for any inconvenience caused.

